# De Nova York para o Rio de Janeiro: a viagem de um livro, 1925

**DOI:** 10.1590/S0104-59702025000100049

**Published:** 2025-11-03

**Authors:** Heloísa Helena Pimenta Rocha

**Affiliations:** i Professora, Faculdade de Educação/Universidade Estadual de Campinas. Campinas – SP – Brasil heloisah@unicamp.br

**Keywords:** Educação sanitária, livros ilustrados, história transnacional, Health education, Illustrated books, Transnational history

## Abstract

Os intercâmbios entre o Brasil e os EUA em torno da educação sanitária das crianças responderam, nas décadas iniciais do século XX, pela circulação de sujeitos, saberes e impressos, envolvendo acordos entre órgãos governamentais de saúde pública, agências internacionais e organizações da sociedade civil. Este artigo apresenta fonte significativa para a compreensão dos processos de produção e circulação transnacional de impressos destinados ao ensino de noções de higiene e saúde: o *Alfabeto da saúde da criança*, livro ilustrado em formato de abecedário, publicado em Nova York e adaptado para o português por J.P. Fontenelle, médico-educador que realizou estágio na Universidade Johns Hopkins, com bolsa da Fundação Rockefeller.

## Abstract (en)

Exchanges between Brazil and the United States regarding children’s health education in the early decades of the twentieth century led to the circulation of subjects, knowledge, and printed matters, involving agreements between government public health agencies, international agencies, and civil society organizations. This article presents a significant source for understanding the processes of production and transnational circulation of printed matters aimed at teaching hygiene and health notions: the Child Health Alphabet, an illustrated book in alphabetical format, published in New York and adapted into Portuguese by J.P. Fontenelle, a physician-educator who completed an internship at Johns Hopkins University with a grant from the Rockefeller Foundation.

Os intercâmbios entre o Brasil e os EUA em torno da saúde infantil e, em particular, da educação sanitária das crianças, nas décadas iniciais do século XX, puseram em circulação sujeitos, saberes e impressos, envolvendo acordos entre órgãos governamentais de saúde pública, agências internacionais e organizações da sociedade civil. O intenso fluxo entre os dois países foi marcado por viagens de médicos (alguns deles também professores das escolas normais), educadoras sanitárias e enfermeiras, em cujas bagagens viajaram livros, relatórios e outros registros dos saberes com os quais tiveram contato.

Articulados em torno de objetivos que incluíam a formação especializada em saúde pública e a difusão de novas concepções nessa área, calcadas fundamentalmente na ideia de prevenção das doenças, os intercâmbios entre os dois países, em muitos casos com financiamento da Fundação Rockefeller, resultaram no trânsito de saberes sobre a infância e sua educação. Processo esse favorecido pela aproximação desses profissionais do trabalho desenvolvido por associações voltadas para a saúde infantil e a educação sanitária, bem como pelo contato que estabeleceram com modelos de atuação e publicações destinadas às crianças.

Como parte dos investimentos com vistas a compreender os processos de produção e circulação transnacional de impressos destinados ao ensino de noções de higiene e saúde às crianças, nas escolas primárias ou em outros espaços educativos, este artigo tem como objetivo apresentar uma fonte cujo circuito pode oferecer elementos significativos para o estudo das conexões entre o Brasil e os EUA, em torno da educação sanitária no período: o *Alfabeto da saúde da criança* (Perterson, 1925), um pequeno livro ilustrado, organizado em formato de abecedário.^1^

## Um abecedário chega ao Rio de Janeiro

Em 1925, o Serviço de Propaganda e Educação Sanitária da Diretoria de Saúde Pública do Estado do Rio de Janeiro, em colaboração com a American Child Health Association, publicou a obra *Alfabeto da saúde da criança*. Com autorização da editora The Macmillan Company e impresso nos EUA, o abecedário foi adaptado para o português pelo médico-educador J.P. Fontenelle/José Paranhos Fontenelle (1885-1975) do original de Mrs. Frederick Peterson, publicado em 1918. O exemplar em estudo faz parte do acervo da Biblioteca de História das Ciências e da Saúde da Casa de Oswaldo Cruz/Fiocruz (Coleção Lourival Ribeiro) e contém, em sua página final, um carimbo que o vincula à Diretoria de Saneamento Rural do Serviço de Saneamento Rural, no estado do Rio de Janeiro.

**Figura 1:**
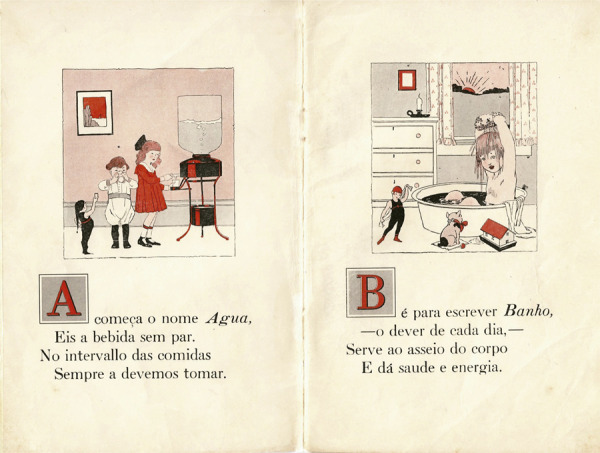
: Lições A e B do abecedário (Peterson, 1925)

O pequeno livro ilustrado, medindo 128x193mm, possui 27 páginas, nas quais são reunidas 25 lições, ordenadas segundo as letras do alfabeto. Na composição da página, que segue o padrão consagrado na produção das obras que davam suporte ao ensino da leitura, a metade superior é ocupada por ilustração alusiva à temática tratada, seguida de prescrições apresentadas em forma de rima, sempre introduzidas por uma letra capitular, como se pode observar nas lições correspondentes às letras A e B (Figura 1).

Destinado a crianças, o livro ilustrado assemelha-se – tanto pelo formato como pelas temáticas abordadas nas lições – a outras publicações que vieram a lume no período, como parte de um conjunto de iniciativas que visava ensinar questões relacionadas a higiene e saúde e, ao mesmo tempo, incutir, desde a mais tenra infância, hábitos considerados saudáveis, entre as quais se incluem a *Cartilha de higiene*, de Antonio de Almeida Junior (1923); a *Fada Hygia*, de Renato Kehl (1936), publicado originalmente em 1925; e as *Rimas para a infância*, de Sara Sampaio Arruda (1927). Exemplar do projeto de educação sanitária em que se inscreve a obra é a lição referente à letra I, na qual se articulam saúde, educação da infância e aquisição de hábitos:

I existe em *Instrucção*,

Servindo bem à saúde

Para criar bons costumes

Que a idade adulta não mude (Peterson, 1925).

**Figura 2:**
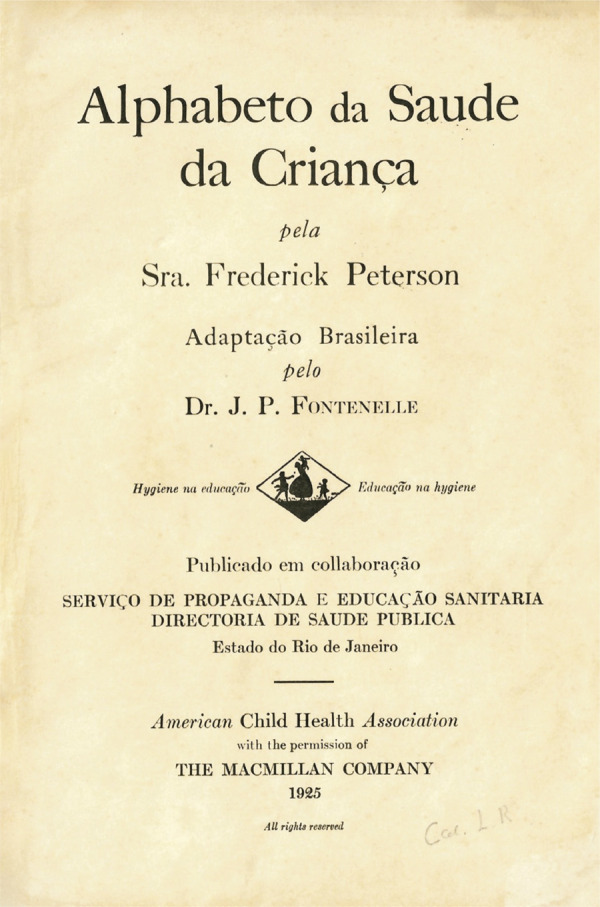
: Folha de rosto do abecedário (Peterson, 1925)

Um aspecto que chama a atenção no exame da obra diz respeito aos seus vínculos com outra obra, da qual ela se configura em uma versão. Indícios desses vínculos aparecem na folha de rosto, em que se pode ler o nome da autora, a indicação de que se trata de uma adaptação para a língua portuguesa, informações sobre as relações de colaboração com a American Child Health Association e com a editora The Macmillan Company, além do distintivo da entidade – a ilustração de uma mulher de perfil erguendo uma criança pequena, cercada por duas outras crianças – acompanhado da divisa que sintetizava as suas iniciativas, “Higiene na educação. Educação na higiene” (Figura 2).

**Figura 3:**
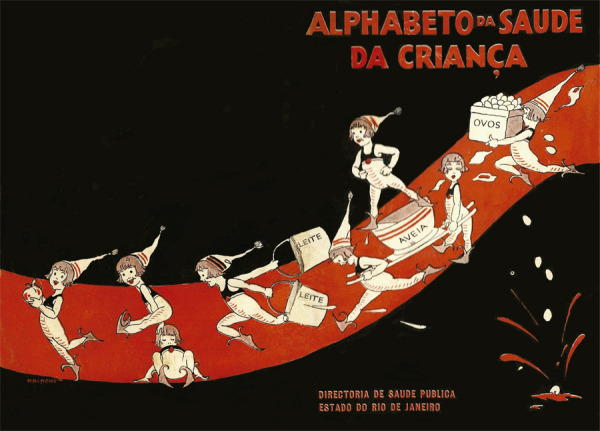
: Capa e quarta capa da obra (Peterson, 1925)

A capa e a quarta capa da publicação (Figura 3), trabalhadas em continuidade, como imagem única, ilustrada em tons de vermelho, branco e preto, colocam em cena figuras femininas representadas como seres fantásticos, vestidas todas com traje igual, usando botas e, algumas delas, gorros. Percorrendo uma espécie de caminho, traçado em vermelho sobre fundo preto, as figuras parecem personificar, de forma lúdica, os hábitos que se pretendia difundir entre as crianças, os quais encontram equivalentes nas lições do abecedário. Seja carregando uma grande quantidade de ovos, um pote de aveia, uma fruta, arrastando baldes de leite ou, ainda, correndo, saltitando e descansando, as personagens que ilustram a capa e a quarta capa parecem materializar a alegria e o vigor, que resultariam da saúde conquistada pelas crianças, por meio da aquisição dos hábitos higiênicos a que são apresentadas lição após lição.

**Figura 4:**
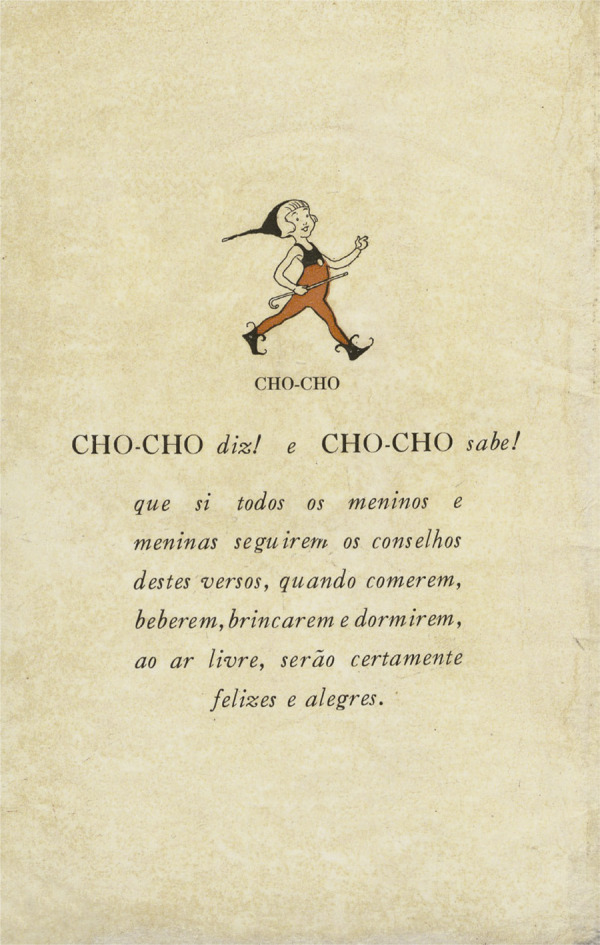
: A fada Cho-Cho, que diz e sabe! (Peterson, 1925)

Uma emblemática figura acompanha as crianças do início ao fim do livro. Presente em todas as lições, a personagem cumpre o papel de apoiá-las nas práticas representadas de asseio corporal, alimentação, exercícios físicos e repouso, mas também de marcar as interdições, como o uso dos xaropes ou o consumo do chá e do café. Nesse sentido, a fada Cho-Cho, como é nomeada, atravessa, a largos e decididos passos, o pequeno livro, afirmando, mesmo antes do início das lições, sua convicção nas certezas da higiene com as quais as crianças se defrontariam nesse percurso de orientações, que vai do A ao Z. Assim, pode-se ver a personagem, convicta, bastão em punho, a afirmar a importância de seguir suas orientações para alcançar uma vida feliz e alegre (Figura 4).

Representada da mesma forma que no texto inicial, a personagem reaparece ao final das lições, dessa vez, para convidar os pequenos leitores a acompanhar o seu próprio crescimento, com base em uma tabela na qual se registram a estatura e o peso esperados de crianças entre os 5 e os 14 anos, sem distinção de gênero. À tabela, seguem-se informações sobre o peso que essa genérica criança deveria ganhar por mês, segundo três grupos de idade: 5-8, 9-11 e 12-14 anos. Dessa vez, afirma a fada:

CHO-CHO deseja saber o peso

de cada criança

E

CHO-CHO deseja saber quanto cada criança aumentou no mês (Peterson, 1925).

Em sua última aparição, a personagem se dirige às crianças, lançando mão de um imperativo: “Aumente o mais que for possível” (Peterson, 1925). Enunciado que, em conjunto com as lições sobre alimentos (ovos, cereais, vegetais, frutas, pão, manteiga e leite) e a tabela para o acompanhamento do ganho de peso e altura, evidencia a centralidade assumida pelas questões ligadas à alimentação, em suas relações com o crescimento das crianças, sinalizando; além disso, a responsabilidade que se esperava que as próprias crianças assumissem quanto à aquisição dos hábitos prescritos. Chamam a atenção as alusões à alegria e ao prazer associados à saúde, expressos em rimas e ilustrações, como se pode observar, por exemplo, na lição correspondente à letra G, de Ginástica, que “dá força, prazer, saúde” e da letra T, de Tigela, “onde vos vem mais comida. Seja leite ou seja sopa. Com prazer é recebida” (Peterson, 1925).

Não se pode desconsiderar os parentescos do abecedário com outras publicações voltadas para a propaganda e a educação sanitárias que circularam no período. Exemplar, nesse sentido, são os impressos produzidos no Instituto de Higiene de São Paulo, no âmbito da reforma sanitária instituída por Geraldo Horácio de Paula Souza, em 1925 (Rocha, 2003), e os elaborados sob orientação de Antônio Luis Cavalcanti de Albuquerque de Barros Barreto, para dar suporte à difusão dos preceitos higiênicos na reforma sanitária implementada na Bahia, entre 1924 e 1930 (Batista, out.-dez. 2019). Não é demais lembrar que ambos, Paula Souza e Barros Barreto, realizaram estágio na Escola de Higiene e Saúde Pública da Universidade Johns Hopkins, com bolsa da Rockefeller, e eram entusiastas dos métodos de educação sanitária aprendidos nos EUA, os quais apelavam a diversos métodos de propaganda sanitária com vistas à formação da consciência sanitária, entre os quais aqueles que se baseavam na palavra escrita (Batista, out.-dez. 2019). Interessa aqui, no entanto, assinalar os vínculos dessa obra com o abecedário publicado em 1918, nos EUA, sob os auspícios da Child Health Organization of America, cujo acrônimo, conforme afirmava o médico-educador brasileiro Carlos Sá, teria dado origem ao nome da fada Cho-Cho.

## Um abecedário é publicado em Nova York

A edição original da obra adaptada para o português, publicada em 1918, sob o título *Child health alphabet* (Peterson, 1922), mede 127x187mm, possui 31 páginas e reúne 26 lições (incluindo a letra W, ausente na versão em língua portuguesa).^2^ A capa e a quarta capa seguem o projeto gráfico mantido na adaptação da obra para o português. O conjunto de lições é seguido de duas tabelas, elaboradas pelo médico Thomas Wood, contendo medidas de altura e peso, conforme a idade, uma para meninas e outra para meninos, abrangendo a faixa dos 5 aos 18 anos de idade, distintamente da versão adaptada, que unificou as medidas em uma só tabela. Cada tabela é acompanhada de uma informação sobre o ganho de peso esperado por mês, organizada em cinco grupos etários: 5-8, 8-11, 11-14, 14-16 e 16-18 anos. Uma página final compila um conjunto de dados sobre a Child Health Organization, seus membros e suas iniciativas editoriais voltadas para as crianças em idade escolar. Esse espaço divulga ainda o acordo estabelecido com a editora Macmillan, com vistas à publicação da série de livros sobre questões ligadas à saúde produzidos pela entidade, entre os quais estava o abecedário que viria a ser adaptado pelo médico-educador brasileiro.^3^

Em 1919, o Department of Home Economics da Universidade de Chicago divulgou uma lista de livros recém-publicados para o trabalho com economia doméstica, na qual é possível encontrar algumas informações sobre a Child Health Organization, bem como sobre o livro de Peterson, em momento muito próximo ao lançamento da primeira edição. Em relação à entidade, o informe assinalava a sua atuação na produção de um material significativo para uma campanha de educação sanitária entre as crianças, o qual incluía uma série de reimpressões versando sobre a desnutrição, pequenos folhetos produzidos para os trabalhos da própria organização, além de etiquetas e cartões, que poderiam ser utilizados nos trabalhos em clínicas de nutrição (Recent Books..., abr. 1919, p.317). Entre os livros dignos de nota, figurava o abecedário produzido por Peterson, sobre o qual se afirmava:

Um ABC ilustrado que começa com ‘A é para maçãs [Apples] e também para Ar’, e segue pelo alfabeto com sugestões de saúde para cada letra. As imagens são atraentes e as rimas – exceto algumas obviamente escritas apenas para completar o alfabeto – são boas e diretas. Isso poderia ser muito útil no trabalho com crianças pequenas.

Entre os outros materiais, estão um cartaz de parede para uso na sala de aula, um cartão de altura e peso para o menino ou a menina levar no bolso, e um marcador com a altura e o peso da criança, além da indicação do seu ganho normal por mês (Recent Books..., abr. 1919, p.318).^4^

A autora do pequeno abecedário era a escritora de livros infantis Antoinette Rotan Peterson (1871-1959), que se assinava Mrs. Frederick Peterson, em referência ao nome do seu marido, um médico que, à época da publicação, fazia parte da junta diretiva da Child Health Organization, respondendo pela secretaria. Uma nota publicada por ocasião do falecimento da autora (Recent Deaths, 5 jun. 1959) dá conta de que ela foi uma das fundadoras, além de tesoureira da entidade, tendo trabalhado com o reconhecido médico L. Emmett Holt, especialista em doenças infantis, que ocupava, quando da publicação da obra, o cargo de presidente da organização.

## Entidades estadunidenses publicam impressos

No exame dos meandros da viagem desse livro entre Nova York e o Rio de Janeiro, é importante indagar sobre as entidades estadunidenses envolvidas em sua publicação/adaptação e suas iniciativas voltadas para a preservação da saúde infantil, as quais incluíam a produção de impressos com vistas à difusão de preceitos higiênicos. Nessa direção, um artigo veiculado na revista mensal *Vers la Santé*, publicada em Paris pela Ligue des Sociétés de la Croix-Rouge, pode oferecer algumas pistas. Na seção intitulada “Higiene no mundo”, o periódico pôs em circulação, na edição de dezembro de 1922, o texto “L’Hygiéne de l’enfance aux États-Unis”, no qual se pode ler: “Uma das poderosas associações de saúde infantil nos EUA, a Child Health Organization of America, há muito se especializou em impressos de propaganda, empregando recursos da ciência e da criatividade” (L’Hygiene..., dez. 1922, p.574).

A Child Health Organization, que teve sua origem ligada à Academia de Medicina de Nova York, segundo informava o artigo, respondia a solicitações vindas de particulares, grupos ou serviços públicos de saúde infantil. Tendo como lema “saúde em educação; educação em saúde” (“*health in education; education in health*”), a entidade se voltava para as crianças em idade escolar, procurando despertar o interesse público pela sua saúde e incentivar o ensino da higiene nas escolas, com o objetivo de inculcar-lhes hábitos saudáveis desde a infância. Referindo-se aos dois eixos que compunham o lema da organização, o artigo sinalizava que “não é exagerado dizer que é em grande parte graças a seus esforços que as escolas primárias americanas tendem cada vez mais a realizar esse duplo propósito” (L’Hygiene..., dez. 1922, p.575).

Examinando a emergência de programas voltados para a saúde da criança, após a Primeira Guerra Mundial, Rosen (1994) registra a criação da entidade, que tinha em sua direção um conhecido pediatra, L. Emmett Holt, e a enfermeira Sally Lucas Jean. Conforme aponta, “mais do que apenas advertir contra as doenças, eles acentuavam o potencial da promoção da saúde por meio da educação e da nutrição. Como a organização agisse, principalmente, nas escolas e se interessasse, primariamente, pelas crianças, introduziram-se em seu trabalho notas de jovialidade e humor” (p.302). Como exemplo dessas “notas de jovialidade e humor”, o autor faz alusão ao abecedário com suas “ilustrações atraentes”.

Os vínculos da entidade com os órgãos estatais se evidenciam quando se observa que o programa destinado ao público escolar resultara, segundo noticiava a *Vers la Santé*, em uma publicação amplamente distribuída pelo Departamento Federal de Instrução Pública dos EUA. Documento esse que compilava o conjunto de aspectos a serem observados com vistas a alcançar o objetivo de preservar a saúde das crianças, contemplando, entre outros, o exame físico e mental antes do ingresso na escola, o controle frequente do peso e da altura, a inspeção de asseio, além de todo um conjunto de práticas que visavam à inculcação de hábitos higiênicos, tarefa considerada infinitamente mais importante que um curso teórico sistemático de higiene. Para tanto, recomendava-se aproveitar todas as ocasiões que se apresentassem na escola, bem como lançar mão do concurso dos pais e das associações ligadas à higiene (L’Hygiene..., dez. 1922).

O artigo veiculado na revista francesa registrava também a atuação da American Child Hygiene Association, entidade criada em 1909, voltada para questões ligadas à saúde materno-infantil. Inicialmente nomeada como American Association for the Study and Prevention of Infant Mortality, a entidade tinha sua atuação orientada para a redução da mortalidade infantil, em articulação com a difusão dos preceitos higiênicos. Nesse sentido, seu presidente, Herbert Hoover, ressaltava a importância do ensino de aspectos ligados ao nascimento e à criação das crianças, sem perder de vista a iniciação nos conhecimentos relacionados à higiene: “Nosso objetivo ... é que toda criança americana nasça em boas condições; seja criada em um ambiente higiênico; receba alimentação adequada, seja constante e cuidadosamente monitorada quanto à saúde e tratada nos primeiros sintomas de doenças; seja iniciada em higiene desde tenra idade” (L’Hygiene..., dez. 1922, p.575-576).

Essas duas entidades passaram a integrar, em 1920, o National Child Health Council, redundando do trabalho em comum nesse órgão a decisão de fundi-las, a partir de 1923. Tal fusão deu origem à American Child Health Association, que, em cooperação com o Serviço de Propaganda e Educação Sanitária, publicou a versão do abecedário em português. Como informava o artigo, a Child Health Organization já acumulava, a essa altura, ampla experiência na produção de impressos destinados às crianças: “Há alguns anos, ela publicou uma notável coleção de cartazes extraordinariamente expressivos. Agora, está distribuindo material para uso diário da própria criança, de modo a utilizar o tempo e o trabalho escolar para familiarizá-la com as noções que desejamos lhe incutir” (L’Hygiene..., dez. 1922, p.574).

Anne Whitney (1929) registra que o serviço educacional da nova entidade, a American Child Health Association, promovia reuniões de alcance nacional para discutir os problemas de saúde na escola, mantendo estreita cooperação com outros grupos nacionais que atuavam nas áreas de educação e saúde, organizando-se, desse modo, como uma central de intercâmbio de informações sobre ideias e materiais para os profissionais dessas áreas. Nas atividades realizadas em escolas e associações, a entidade que a antecedeu (CHO) lançava mão, segundo informava a revista francesa, não apenas dos impressos, mas de um palhaço, uma fada e um ventríloquo, o que provavelmente dava ensejo à encenação das peças reunidas nos livros publicados por aquela associação.^5^ Tais encenações são lidas por Rosen (1994, p.303) como tentativas que, apesar de serem superficiais e acentuarem excessivamente “os aspectos ‘radiantes’ da saúde”, tiveram importante papel no deslocamento do caráter visual das atividades anteriores, que o autor caracteriza como “repulsivo”.

Não deve parecer casual a veiculação do artigo sobre essas entidades estadunidenses e suas iniciativas na revista francesa publicada pela Cruz Vermelha, quando se leva em conta as articulações internacionais em torno da saúde infantil e, de modo mais específico, da difusão das noções de higiene e saúde para as crianças, que ganham visibilidade no período. Ethel Perrin, diretora associada da American Child Health Association, responsável pelo serviço educacional da entidade, informa, por exemplo, a iniciativa de concessão de uma bolsa pela American Association of Teachers Colleges, em cooperação com a Metropolitan Life Insurance Company e a American Child Health Association, para o desenvolvimento de estudos na Europa, com o propósito de aprimorar o trabalho dos profissionais que se dedicavam à formação dos professores em temas de educação sanitária, num momento em que vários dos profissionais mais reconhecidos no campo já haviam sido contemplados com bolsas oferecidas durante os primeiros anos de atuação da Child Health Organization (Perrin, 1933, p.3).^6^ Compilando as ações levadas a cabo por essas entidades, Rosen (1994) ressalta seu papel no reconhecimento da educação em saúde como um campo específico. Nessa direção, faz referência a iniciativas como a conferência convocada pela Child Health Organization, em 1919; a concessão de uma bolsa de estudos oferecida por essa mesma entidade no ano seguinte; bem como a conferência organizada pela American Child Health Association, em colaboração com o Departamento de Educação dos EUA, na qual se enfatizou a necessidade de formação de professores em questões de saúde.

O texto publicado na *Vers la Santé* apresenta uma lista de impressos publicados pela Child Health Organization, no período anterior à fusão que resultou na criação da American Child Health Association, entre os quais figuram, além da obra adaptada para o português por Fontenelle, cadernos de desenho para colorir, contos de fadas ilustrados e livros de poemas sobre temáticas ligadas à higiene e saúde (L’Hygiene…, dez. 1922, p.574). Os dados levantados permitem observar que o abecedário se articula a uma série de iniciativas voltadas para a educação sanitária das crianças, promovidas por entidades estadunidenses envolvidas com a saúde e a educação, entrecruzando-se com os percursos de formação em saúde pública financiados pela Fundação Rockefeller.

## Um médico-educador brasileiro viaja aos EUA

O exame de alguns aspectos da trajetória de J.P. Fontenelle, médico-educador responsável pela adaptação do *Child health alphabet*, permite uma aproximação dos caminhos percorridos pela obra até sua publicação em língua portuguesa, bem como das conexões internacionais em torno da saúde infantil, do ensino de higiene para crianças e da formação especializada e atuação em saúde pública, segundo novos parâmetros, de que a obra é um dos indícios.

Cabe assinalar, em primeiro lugar, que Fontenelle era formado pela Faculdade de Medicina do Rio de Janeiro (1908), tendo iniciado sua atuação como inspetor sanitário interino da Diretoria Geral de Saúde Pública do Rio de Janeiro, em 1909, cargo no qual foi efetivado em 1914. Em 1917, tornou-se professor de Higiene da Escola Normal do Distrito Federal, publicando, no ano seguinte, a obra *Compêndio de higiene elementar* (Fontenelle, 1925), na qual sistematizava os conhecimentos abordados na formação das normalistas (Paiva, 2013). Fontenelle era membro da Sociedade de Medicina e Cirurgia do Rio de Janeiro (SMCRJ), na qual ingressou em 1921. No campo educacional, foi membro da Associação Brasileira de Educação, figurando entre os signatários do Manifesto dos Pioneiros da Educação Nova, em 1932.

Em relação aos contatos que franqueariam a adaptação da obra, é importante atentar para o fato de que, segundo Castro Santos e Faria (2006, p.324) ele foi “bolsista da Fundação Rockefeller, entre outubro de 1925 e abril de 1926, na Escola de Higiene e Saúde Pública da Universidade Johns Hopkins”, escola que se constituiu na “primeira instituição educacional apoiada pelo projeto Rockefeller” (Batista, out.-dez. 2019, p.1190), atuando como centro de formação de profissionais de saúde de vários países do mundo (Batista, Ferreira, maio-ago. 2021). Assim, o contato de Fontenelle com as iniciativas de associações como a American Child Health Association, que resultou na publicação do abecedário ilustrado em língua portuguesa, pode ter se dado no contexto do estágio de formação desse inspetor sanitário brasileiro, professor da Escola Normal do Distrito Federal, em um dos mais importantes centros de formação em saúde pública no período, para o qual foi escolhido por meio de processos de seleção que passavam pela ingerência dos grupos locais e por apostas nos postos de comando que ele viria a assumir em seu retorno, como se pode depreender das análises de Batista e Ferreira (maio-ago. 2021) sobre os meandros da seleção de bolsistas enviados aos EUA entre 1919 e 1924.

Destino de bolsistas como Fontenelle, a Escola de Higiene e Saúde Pública da Universidade Johns Hopkins foi criada em 1916, com o objetivo de formar mão de obra para atuar em saúde pública, contando com financiamento da Fundação Rockefeller, sociedade civil sem fins lucrativos, criada nos EUA, em 1913, a par da reorganização das juntas filantrópicas patrocinadas pela milionária família Rockefeller, desde o final do século XIX. A escola tornou-se referência para a formação especializada na área, servindo de “modelo para instituições semelhantes criadas com o apoio da Fundação Rockefeller em todo o mundo” (Marinho, 2001, p.24-25). Como assinala Souza (2012, p.21), a concessão de bolsas de estudo pela Fundação Rockefeller foi decisiva na formação de uma “nova geração de sanitaristas”, que, em seu retorno ao país, trouxe uma concepção de saúde calcada na profilaxia das doenças infectocontagiosas e nos métodos experimentais.

Nesse sentido, a formação especializada nos EUA, com bolsa da Fundação Rockefeller, não era algo incomum entre os quadros da saúde pública, no período, constituindo-se a passagem pela Johns Hopkins um ponto de interseção na trajetória de muitos dos profissionais que ocupariam cargos importantes nos órgãos estatais (Batista, out.-dez. 2019; Batista, Ferreira, maio-ago. 2021; Korndörfer, jan.-jun. 2016). Conforme apontam Castro Santos e Faria (2006, p.293), “os médicos do Brasil que se formaram em saúde pública na Universidade Johns Hopkins alargaram os caminhos para o surgimento de um novo modelo de atuação profissional, sustentado na educação sanitária, na prevenção de doenças e na formação de recursos humanos”.

A ficha individual de J.P. Fontenelle produzida pela Fundação Rockefeller, reunindo informações sobre sua trajetória como bolsista, registra que, além de realizar o curso de administração em saúde pública e desenvolver trabalhos em epidemiologia, interessava a ele passar dois meses visitando organizações de saúde estaduais, municipais e distritais, com o objetivo de adquirir experiência prática, o que parece ter se concretizado, pelo menos em parte, com a série de visitas realizada durante a segunda quinzena de março de 1926, quando ele percorreu Montgomery, Andalusia e Birmingham (Alabama). O falecimento do seu pai responderia pelo seu retorno ao Brasil antes do período inicialmente previsto. Quanto à presença de Fontenelle no cenário internacional, cabe notar que seus vínculos com os EUA não se limitaram ao período em que foi bolsista da Rockefeller, já que, anos mais tarde, em 1948, ele foi eleito vice-presidente da Associação Americana de Saúde Pública.

As preocupações do médico-educador com o ensino de higiene nas escolas primárias já se faziam observar antes mesmo da sua viagem aos EUA. Tematizadas no compêndio produzido no contexto da sua atuação na formação de normalistas, elas eram também objeto das discussões que tinham lugar na SMCRJ, como evidenciam seus pronunciamentos nessa corporação. Suas reflexões em relação ao tema e a defesa do caráter exemplar das iniciativas em curso nos EUA eram partilhadas por outros médicos que também ocupavam a tribuna da sociedade, como Carlos Sá, formado pela Faculdade de Medicina do Rio de Janeiro (1907), integrante dos quadros do Departamento Nacional de Saúde Pública (DNSP), a partir de 1921, e, assim como Fontenelle, professor de Higiene da Escola Normal do Distrito Federal (1923), que também viajou aos EUA para aperfeiçoar sua formação em saúde pública.

Em conferência proferida na SMCRJ, sob o título “O ensino da higiene nas escolas primárias”, Sá (jul. 1922) destacava a exemplaridade das iniciativas da Child Health Organization, calcadas no reforço positivo dos gestos que se esperava que fossem adotados pelas crianças. Nesse sentido, referindo-se aos projetos por meio dos quais se poderia realizar o ensino da higiene e da saúde na escola, registrava o recurso às dramatizações de que lançavam mão os estadunidenses. Na obra *Higiene e educação da saúde*, que publicou anos mais tarde, como resultado de um curso ministrado às professoras primárias entre 1941 e 1942, assinalaria: “Entre as ‘dramatizações’, citam-se a Fada da Saúde que vem contar histórias às crianças e o Palhaço Chô Chô que faz graças de interesse higiênico (C.H.O. são as iniciais de Child Health Organization, isto é, traduzindo, Organização de Saúde da Criança)” (Sá, 1952, p.238-239; destaque no original).

No exame dos meandros do processo que respondeu pela adaptação do abecedário ilustrado para a língua portuguesa e por sua publicação com o selo de um órgão estatal, é importante ter em conta, para além do estágio de Fontenelle na Johns Hopkins como bolsista da Fundação Rockefeller e dos seus possíveis contatos com entidades como a American Child Health Association, Brasil, com o selo de um órgão estatal, o cenário nacional e as reconfigurações que redundaram na criação do Departamento Nacional de Saúde Pública, em 1920. Órgão subordinado ao Ministério da Justiça e Negócios Interiores, o DNSP resultou de um amplo movimento político de caráter nacionalista, que teve lugar na Primeira República, como sublinham os estudos de Castro Santos (1985) e Hochmann (1998), cujas reflexões são fundamentais quando se busca compreender os impactos do movimento sanitarista na constituição de uma agenda política nacional de saúde pública e saneamento, bem como na implementação dos arranjos legais e institucionais para a sua viabilização, em articulação com o processo de construção do Estado.

A centralidade assumida pela saúde pública no debate político nacional, como um dos efeitos desse movimento, no âmbito do qual a superação das doenças endêmicas figurava como condição para a constituição da nacionalidade, responderia pela criação desse órgão, calcado nos intentos de unificação e centralização dos serviços de saúde pública, materializando, por essa via, a ampliação do poder do Estado nessa área. Entre as esferas de ação que passaram a ser assumidas pelo DNSP, destaca-se a responsabilidade pela educação da população em questões ligadas à saúde, expressa na criação do Serviço de Propaganda e Educação Sanitária, em 1923, subordinado à Diretoria Geral do DNSP, o qual tinha como principal objetivo promover a divulgação das noções de higiene pessoal e pública, como assinala Souza (2012). Atento ao que estava sendo desenvolvido em outros países, no período, quer nas esferas estatais, quer no âmbito de associações particulares, esse serviço imprimiu às suas produções um viés marcadamente estadunidense, o que pode ser compreendido quando se leva em conta os investimentos na formação profissional dos quadros da saúde pública na Johns Hopkins, com financiamento da Rockefeller.

Nesse sentido, os contatos dos médicos brasileiros, neste caso médicos-educadores, com as entidades estadunidenses que produziram impressos como o abecedário, adaptado para o português por J.P. Fontenelle, não se constituíram em mero fruto do acaso, apontando para a importância de se interrogar sobre os vínculos entre o Serviço de Propaganda e Educação Sanitária do DNSP, a American Child Health Association, a The Macmillan Company, a Fundação Rockefeller e os órgãos oficiais de saúde pública estadunidenses. Quanto a esses vínculos, o estudo de Paiva (2013, p.34) faz alusão a uma “colaboração financeira e técnica de uma organização norte-americana” na produção do *Alfabeto da saúde da criança*, embora não ofereça detalhes que permitam dimensionar a natureza e o montante dessa colaboração. Assim, apesar das lacunas em relação a esse abecedário e ao seu trânsito entre Nova York e o Rio de Janeiro – expressas, por exemplo, na falta de informações sobre o ilustrador, o contrato de edição da versão em português, o processo de adaptação, a tiragem e a distribuição –, cabe assinalar a importância dessa fonte para as investigações sobre a circulação de sujeitos, saberes e impressos entre o Brasil e os EUA e o peso destes intercâmbios na definição de políticas de educação sanitária no contexto dos projetos de modernização do país (Birn, jul.-set. 2007), bem como no desenho das iniciativas por meio das quais se visou ensinar às crianças e suas famílias novas formas de viver a vida cotidiana.

## NOTAS

1 O livro ilustrado integra o *corpus* documental de uma pesquisa em desenvolvimento, financiada pelo Conselho Nacional de Desenvolvimento Científico e Tecnológico (Bolsa de Produtividade em Pesquisa), sob o título “Educação sanitária entre Brasil e Estados Unidos: circulação de saberes, sujeitos e objetos”, e pela Fundação de Amparo à Pesquisa do Estado de São Paulo, no âmbito do projeto temático “Saberes e práticas em fronteiras: por uma história transnacional da educação (1810- )”.

2 A edição utilizada, publicada em 1922, faz parte do acervo da University of California, disponível na HathiTrust Digital Library.

3 Fundada em Londres em 1843, a Macmillan abriu sua primeira filial nos EUA em 1869, inicialmente como uma agência de distribuição. Em 1886, deu início à publicação de livros, permanecendo como propriedade da Macmillan London até 1951 e mantendo com esta um acordo de reciprocidade, que assegurava a publicação dos livros em ambos os países. Os dados sobre a editora estão disponíveis no Macmillan & Co. Ltd. Archive: https://collections.reading.ac.uk/special-collections/collections/macmillan-and-co-ltd-archive.

4 Nesta e nas demais citações de textos em outros idiomas, a tradução é livre.

5 Ethel Perrin (1933) faz alusão aos métodos utilizados pela Child Health Organization para despertar também o interesse e o entusiasmo dos professores e administradores pela educação sanitária, entre os quais destaca os resultados do *Cho-Cho and the Health Game* para a construção de um programa de educação das crianças, calcado no autogoverno em questões relacionadas à saúde individual e coletiva.

6 Referindo-se às iniciativas em curso nos EUA, *Vers la Santé* informava que, durante o período em que as duas entidades que deram origem à American Child Health Association estavam vinculadas ao National Child Health Council, esse órgão realizou uma exposição nacional de proteção à mãe e à criança em Mansfield e Richland (Ohio), com recursos doados pela Cruz Vermelha, o que aponta para os vínculos entre essas entidades, órgãos governamentais e agências que atuavam em escala global, como era o caso da Cruz Vermelha.
